# Biological assessment of new tetrahydroacridine derivatives with fluorobenzoic moiety in vitro on A549 and HT-29 cell lines and in vivo on animal model

**DOI:** 10.1007/s13577-020-00376-0

**Published:** 2020-05-24

**Authors:** Karol Kłosiński, Małgorzata Girek, Kamila Czarnecka, Zbigniew Pasieka, Robert Skibiński, Paweł Szymański

**Affiliations:** 1 Department of Experimental Surgery, Faculty of Medicine, Medical University of Lodz, Narutowicza 60, 90-136 Lodz, Poland; 2 Department of Pharmaceutical Chemistry, Drug Analysis and Radiopharmacy, Faculty of Pharmacy, Medical University of Lodz, Muszyńskiego 1, 90-151 Lodz, Poland; 3 Department of Medicinal Chemistry, Faculty of Pharmacy, Medical University of Lublin, Jaczewskiego 4, 20-090 Lublin, Poland

**Keywords:** Acridine derivatives, Colorectal cancer, Cytotoxicity, Lung cancer, MTT assay

## Abstract

A new series of tetrahydroacridine derivatives with the fluorobenzoyl moiety was synthesized and evaluated for cytotoxic activity against lung cancer cell lines A549 and colorectal cancer HT29. The cytotoxic activity of the compounds was compared on the somatic cell line—EAhy926. Compounds showed high cytotoxic activity on A549 cells (IC_50_ 183.26–68.07 μM) and HT29 cells (IC_50_ 68.41–19.70 μM), higher than controls—etoposide (IC_50_ 451.47 μM) toward A549 and 5-fluorouracil (IC_50_ 1626.85 μM) against HT29. Derivative **4** was the most cytotoxic to A549, whereas for the cell lines HT29 compound **6**. Selected compounds showed similar cytotoxicity to the EAhy926 cell line (IC_50_ about 50 μM). In the hyaluronidase inhibition assay, all compounds exhibited anti-inflammatory activity, including **4** exhibiting the best inhibitory activity—IC_50_ of 52.27 μM when the IC_50_ heparin was 56.41 μM. Mathematical modeling was performed to determine LD_50_ after intraperitoneal, oral, intravenous and subcutaneous administration and to predict potential mutagenicity and carcinogenicity of the compounds analyzed. Obtained results showed that tested derivatives are slightly toxic compounds, and LD_50_ values (mg/kg) ranged from 680 to 1200 (oral rat model), the analyzed compounds have low mutagenic potential, and differences between derivatives are insignificant and very low probability of carcinogenicity. To confirm mathematical calculations, an in vivo test was carried out on a laboratory mouse model for two selected compounds. It allowed to qualify compounds: **6** to category 4 of the GHS scale, and **4** to category 3 of the GHS scale.

## Introduction

Cancer disease regards abnormal cell growth, which can further spread to other parts of the body and metastasize. Around 100 million people suffer from cancer worldwide. In 2018, there was about 18.1 million new cancer cases, which caused 9.6 million cancer deaths [1]. Cancer treatment costs 1.16 trillion USD per year. The most common types of cancer are lung, prostate, colorectal and stomach cancer in men, whereas breast, colorectal, lung and cervical cancer appear mostly in women [2]. People live longer and they have changed their lifestyle in the developing countries; therefore, new cases of cancer have started to appear more and more widely [3].

Lung cancer is the most common type of cancer in the world, causes 2.1 million new cases and 1.8 million deaths per year [1]. This makes it the most common cause of cancer deaths in men, and second, most often in women [2]. It is a malignant lung tumor, which symptoms regard coughing, weight loss, shortness of breath and chest pains [4]. Lung cancer mostly appear in people who smoke [5], but about 10–15% of cases occur in people who have never smoked [6]. The two main types are small-cell lung carcinoma (SCLC) and non-small-cell lung carcinoma (NSCLC) [7]. Treatment depends on the type of cancer, the stage and the general health of the person. Treatment involves surgery, chemotherapy and radiotherapy. NSCLC is more often treated surgically, while SCLC usually responds better to chemotherapy and radiotherapy [8].

Colon cancer is a malignant cancer, which symptoms may include blood in the stool, a change in bowel movements and weight loss [9]. In the world, colon cancer is the third most common type of cancer, with 1.8 million new cases and 880,000 deaths per year [2]. It occurs much more frequently in developed countries and more often in men than in women [10]. Most colon tumors are associated with bad lifestyle, inadequate diet, aging of the body, and only a small number of cases are caused by genetic disorders [11]. Colon cancer is commonly diagnosed by a sigmoidoscopy or colonoscopy [12]. Treatment of this type of cancer involves a combination of surgery, radiotherapy, chemotherapy and targeted therapy [9].

Acridines have been known as therapeutic drugs for many years and their use regard antibacterial, antiviral and anticancer mechanism of actions. Acridine and its derivatives intercalate into DNA [13] and intercalation process results in unwinding of DNA helix. These actions cause inhibition of replication and transcription, and this mechanism of actions is demanding in cancer treatment [14]. Nowadays one of acridine derivatives—amsacrine—is used as an antineoplastic drug to treat leukemia. Researcher’s team work on novel acridine derivatives, which further can be used in cancer therapy [7, 15–20]. We worked on novel tetrahydroacridine derivatives, which previously had been tested in in vivo biodistribution. Results had showed that compounds accumulated in large amount in the rat’s bowels and lungs [21]. Therefore, novel tetrahydroacridine derivatives were tested for cytotoxicity effect on lung and colon cancer cell lines.

## Materials and methods

### Synthesis of tetrahydroacridine derivatives

The synthesis of new tetrahydroacridine derivatives with fluorobenzo groups has been described previously by Czarnecka et al. [22]. This synthesis consisted of the combination of 9-chloro-1,2,3,4-tetrahydroacridine with 2-fluorobenzoic acid or 3-fluorobenzoic acid using diamine linker. Semi-finished products were used to obtain the final compounds. Reaction of 9-chloro-1,2,3,4-tetrahydroacridine with 2 equivalent of the corresponding a, x-diamine in the presence of phenol at 180 °C and catalytic amounts of the sodium iodide provided these intermediate products in good yield (80–90%). Subsequently, 2-chloro-4,6-dimethyl-1,3,5-triazine (CDMT) was used. The reaction mechanism included the activation of the carboxylic acid group (2-fluorobenzoic or 3-fluorobenzoic acid) by means of CDMT. The reaction may have been carried out in a suitable inert solvent such as dichloromethane, acetonitrile, dioxane or tetrahydrofuran at a temperature below 5 °C. The active ester was obtained, which was then reacted with the appropriate amine, added to the same pot. Compounds were obtained in good yield (about 80–90%), which were then purified by flash chromatography. In the last step, the obtained compounds were converted into the hydrochloride salts by dissolving in a small volume of methanol and adding HCl in ether. The HCl salts of the new compounds were synthesized to achieve higher solubility in polar solvents [22].

### Cell culture

To assess the cytotoxicity of the new compounds, the A549 cell line (lung carcinoma from human) (European Collection of Cell Culture) was selected. Cells were grown in Dulbecco’s Modified Eagle’s Medium (DMEM) (PAN-Biotech), which includes 10% Fetal Bovine Serum (Sigma Aldrich), 2 mM Glutamine (Sigma Aldrich), and 100 units/mL penicillin and 100 mg/ml streptomycin (Sigma Aldrich).

HT29 cell line (colorectal adenocarcinoma from human) (American Type Culture Collection) was chosen to evaluate the cytotoxicity of novel compounds. Cells were grown in McCoy’s Medium (Biological Industries) which includes 10% Fetal Bovine Serum (Sigma Aldrich), 2 mM Glutamine (Sigma Aldrich), and 100 units/mL penicillin and 100 mg/ml streptomycin (Sigma Aldrich).

To compare the cytotoxicity of the new compounds, the EAhy926 cell line (the human umbilical vein, somatic cell hybrid) (American Type Culture Collection) was selected. Cells were grown in Dulbecco’s Modified Eagle’s Medium (DMEM) (PAN-Biotech) which includes 10% Fetal Bovine Serum (Sigma Aldrich), 2 mM Glutamine (Sigma Aldrich), and 100 units/mL penicillin and 100 mg/ml streptomycin (Biological Industries). Before the initiation of the assay, cells were plated and grown in an incubator at 37 °C with 5% CO_2_ to 80% confluence.

### MTT cytotoxicity assay

Cell viability following exposure to synthetic compounds was estimated using the 3-(4,5-dimethylthiazol-2-yl)-2,5-diphenyltetrazolium Bromide (MTT) reduction assay. To perform the MTT test, cells were seeded into 96-well plates at density 10^4^ cells per well and cultured for 24 h at 37 °C and 5% CO_2_. Next the medium was removed and cells were exposed to the 100 μl of the compound solutions over a range of concentrations (final DMSO concentration was below 0.2%) or nothing but culture medium (blank control), and pure DMSO was used as a positive control. Cells with compound solutions were incubated for 24 h. Medium was removed and 50 μl of the MTT solution was added to each well and incubated in the dark for additional 2 h at 37 °C. Later the MTT solution was carefully removed and 100 μl of DMSO was added. Plates were held for 10 min at room temperature. Before placing in a microplate reader (Synergy H1, BioTek, Winooski, VT, USA) 5 μl of Sorensen Buffer was added to each well. Plate was swayed and the absorbance was measured at a wavelength of 570 nm. The cell viability was expressed as a percentage of the control values (blank) [23, 24].

### Hyaluronidase inhibition assay

All compounds were subjected in hyaluronidase inhibition test to determine their inhibitory activity toward enzyme. The hyaluronidase inhibition study was carried out by turbidimetric method modified to the 96-well plates and previously described by Michel et al. [25].

Analyzed compound solutions were prepared freshly right before the assay, and research began by adding 20 μl of the tested compound solution in monosodium phosphate buffer (pH 7.0) and 40 μl of hyaluronidase solution (22.55 U/ml, hyaluronidase from bovine testes Type I-S, Sigma Aldrich) to the wells of 96-well microtiter plates. The solutions were incubated in the dark for 10 min at the temperature 37 ºC and then 40 μl of hyaluronic acid solution (0.03%, Sigma Aldrich) in monosodium phosphate buffer (pH 5.35) was added to the wells. Next, the mixture was incubated in the dark for 45 min at 37 °C. At the end, 300 μl of bovine serum albumin (0.1%, Serva) in sodium acetate buffer (pH 3.75) was added to the wells and incubation was carried out at the room temperature for 10 min.

Using the microplate reader (BioTek, Winooski, VT, USA), the turbidity values were measured. The measurement was made at a length of 600 nM. The assay was run in three experiments in triplicate to calculate the half maximal inhibitory concentration—IC_50_ values. Heparin (WZF, Polfa Warsaw) was a positive control. The inhibitory activity of the tested compounds was calculated as inhibition percentage (% inhibition) of hyaluronidase according to the equation:

$$\% \; {\text{inhibition}} = 100 \times \left( {1 - \left( {\frac{{A_{{{\text{HA}}}} - A_{{{\text{AN}}}} }}{{A_{{{\text{HA}}}} - A_{{{\text{HYAL}}}} }}} \right) } \right) ,$$

where *A*_HA_ is the absorbance of solution without the enzyme (positive control), *A*_HYAL_ the absorbance of solution without the tested compound (negative control), and *A*_AN_ the absorbance of solution with the tested compound.

### In silico toxicity calculations

Acute toxicity to rodents expressed in the dose of compound required to kill half the members of a tested population—LD_50_ values after intraperitoneal, oral, intravenous and subcutaneous administration—was calculated with the use of ACD/Percepta 14.0.0 software (ACD/Labs). The same software was also used for the prediction of the mutagenicity of the analyzed compounds and estimation of the probability of positive Ames test.

The carcinogenicity of the studied tetrahydroacridine derivatives was predicted with the use of Vega 1.1.4 software based on CAESAR 2.1.9 model.

### In vivo acute oral toxicity: acute toxic class method

To determine the mathematical calculations, the effect on living organisms and qualify new tetrahydroacridine derivatives to a specific acute toxicity class, an in vivo test was performed on a laboratory mouse model according to OECD 423 "Acute Oral Toxicity—Acute Toxic Class Method" guidelines. The research was carried out based on the resolution of the Local Ethics Committee for Animal Experiments at the Medical University of Lodz No. 57/115 ŁB/2018.

The chemical substances were given by the oral route in one of four fixed doses (5, 50, 300, 2000 mg/kg) for a given stage of the study. The results will allow you to rank chemicals in commonly used classification systems. According to the diagram from the OECD 423 guidelines, a maximum of 6 test stages can be carried out. Three animals are involved in each stage, and the number of stages depends on the mortality of the animals, which is the final test parameter.

After the quarantine, handling, weighing and labeling, the substance was administered at a dose of 300 mg/kg per body weight (dose used in the absence of information on the toxicity of the test substance). If 2–3 animals are found after using the dose, substances should be given at a lower dose—50 mg/kg (the last dose of 5 mg/kg is used, if after 50 mg/kg, also 2–3 animals die). In the absence of death or death of only 1 animal, the substance is again administered to the stomach at the same dose—300 mg/kg, and in the case of subsequent deaths, a higher dose, 2000 mg/kg, is used. Absence or presence of the mortality of animals exposed at one stage determined the performance of the next stage of the study. After each dose, animals were observed for 14 days. The animals will then be killed by intraperitoneal administration of a lethal dose of sodium pentobarbital.

### Statistical analysis

Values were represented as mean ± SD. One-way ANOVA with post-hoc analysis was made. Statistical analysis was performed in Statistica version 13.1 software.

## Results and discussion

### In vitro cytotoxicity of new compounds against cancer cell lines

**Fig. 1:**
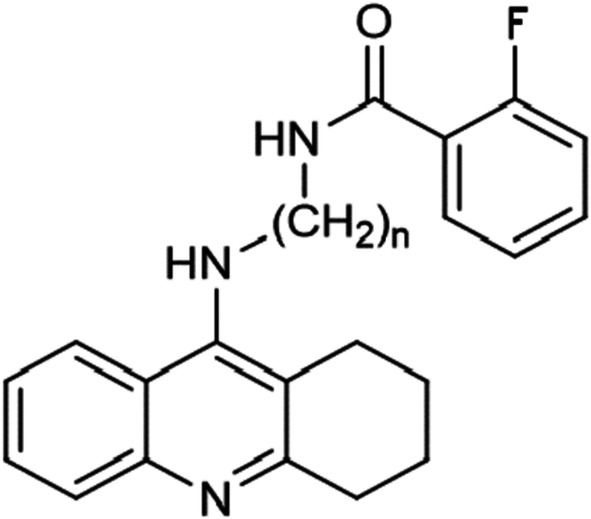
Compound no. **1** (*n* = 2), **2** (*n* = 3), **3** (*n* = 4), substitute: 2-fluorobenzoic acid

**Fig. 2:**
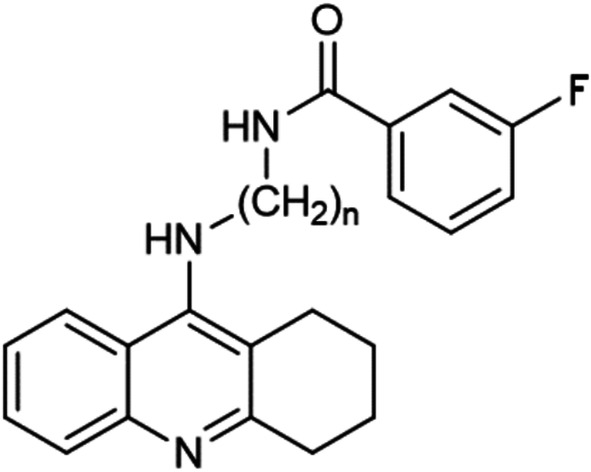
Compound no. **4** (*n* = 2), **5** (*n* = 3), **6** (*n* = 4), substitute: 3-fluorobenzoic acid

Tetrahydroacridine derivatives with the fluorobenzoic acid moiety (Figs. 1, 2) were tested in vitro by the MTT test against two human cancer cell lines—A549 and HT29; and one human non-neoplastic cell line EA.hy926. In cancer cells, p53 tumor suppressor protein plays a crucial role in cancer progression. Between both cancer cell lines, there is a difference between p53. A549 cells possess wild-type p53, whereas in HT-29, there is a mutant p53 (sequence codon change—273)—which results in increased invasion and cell scattering [26]. The IC_50_ value was determined by concentration–response analysis. The results of these tests are presented in Table 1. The positive control of the studies were chemotherapeutic—etoposide and 5-fluorouracil, which are commonly used clinical agents.

**Table 1:** In vitro cytotoxic activity of new compounds and reference compounds on two cancer cell lines (A549 and HT29) and one non-cancer cell line (EA.hy926)

No.	Moiety	Number of methylene groups	IC_50_ [µM] against A549	SD	IC_50_ [µM] against HT29	SD	IC_50_ [µM] against EA.hy9263	SD
**1**	2-Fluorobenzoic acid	2	106.37	± 7.78	68.41	± 2.88	–	–
**2**	2-Fluorobenzoic acid	3	148.20	± 13.96	50.20	± 5.26	–	–
**3**	2-Fluorobenzoic acid	4	183.26	± 4.44	22.98	± 2.03	–	–
**4**	3-Fluorobenzoic acid	2	68.07*	** ± 0.68**	35.30*	± 3.78	52.55	± 7.42
**5**	3-Fluorobenzoic acid	3	117.76	± 9.40	44.02	± 2.34	–	–
**6**	3-Fluorobenzoic acid	4	128.43***	± 13.76	19.70***	** ± 0.52**	50.88	± 3.41
**C1**	Etoposide		451.47	± 18.27	654.03	± 39.51	155.19	± 9.81
**C2**	5-fluorouracil		> 1800		1626.85	± 49.26	> 1800	

All values are presented as the means ± standard deviation (SD)

Compounds 4 and 6 (bold font), as the most cytotoxic, were chosen for the next study

Statistical significance was assessed using one-way ANOVA with a post-hoc analysis was performed

*IC*_*50*_ 50% inhibition of the cell viability, *µM* micromole/liter

***p < 0.001, *p < 0.05 was considered as significantly different between cancer and non-cancer cell line

**Fig. 3:**
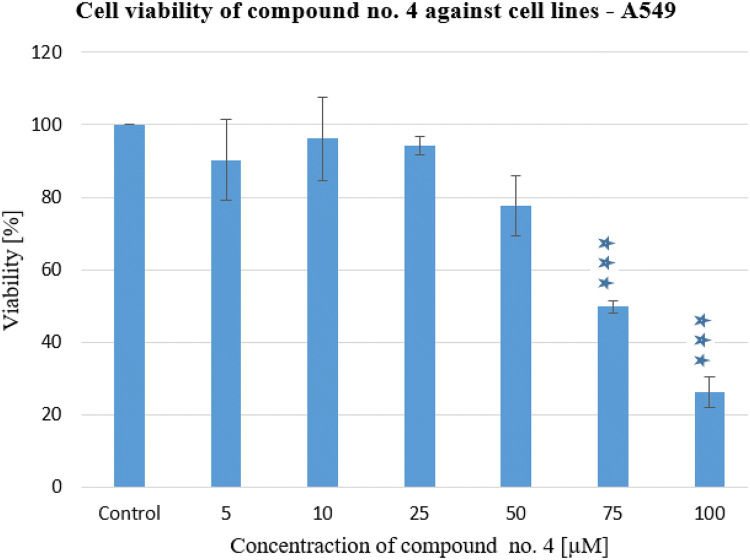
Cell viability of compound no. 4 against cell lines—A549. Experiments were done in triplicates. Statistical significance was assessed using one-way ANOVA with a post-hoc analysis was performed. ***p < 0.001, **p < 0.01, and *p < 0.05 were considered as significantly different in comparison to non-treated control

**Fig. 4:**
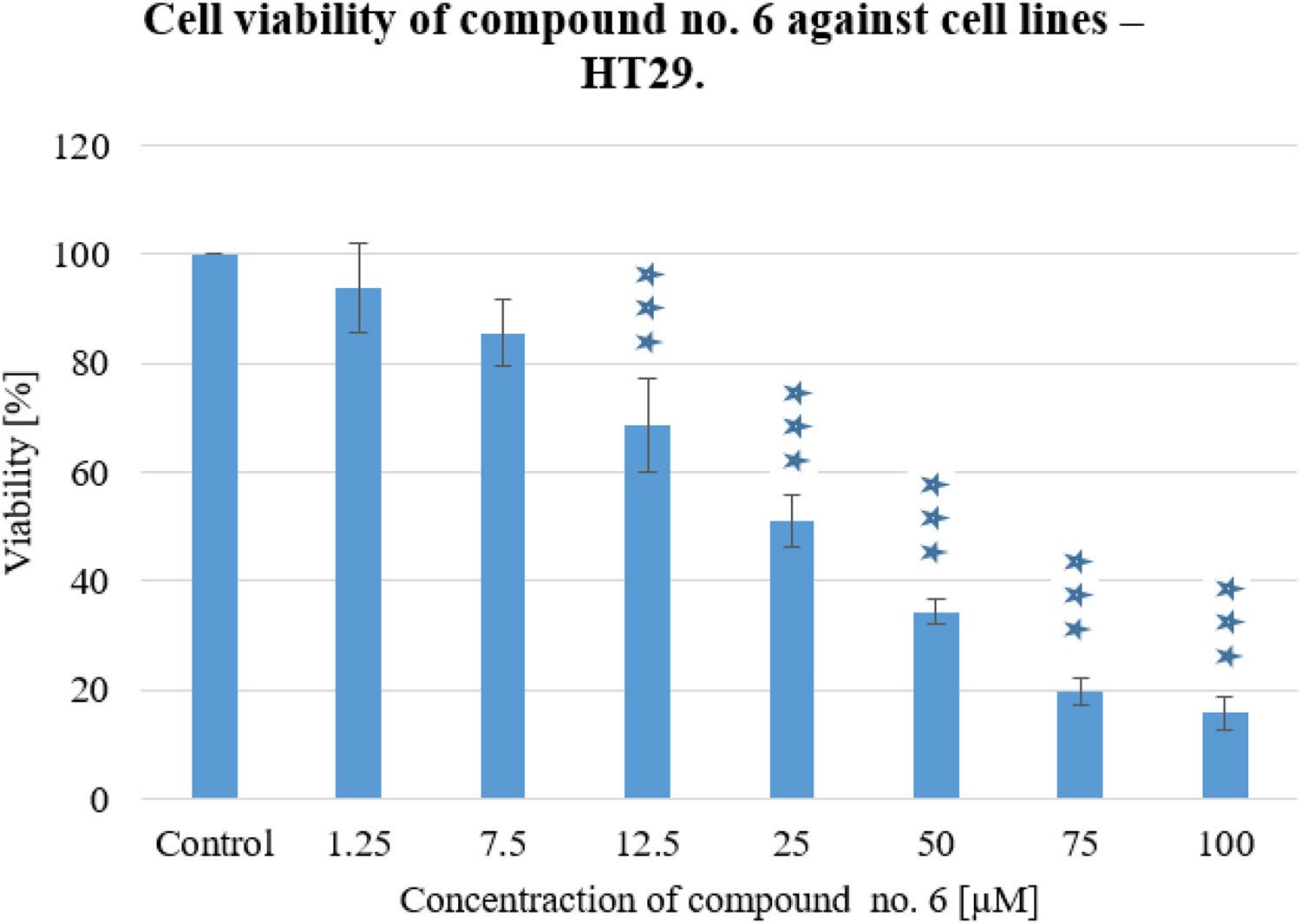
Cell viability of compound no. 6 against cell lines—HT29. Experiments were done in triplicates. Statistical significance was assessed using one-way ANOVA with a post-hoc analysis was performed. ***p < 0.001, **p < 0.01, and *p < 0.05 were considered as significantly different in comparison to non-treated control

All tested tetrahydroacridine derivatives showed cytotoxic activity. IC_50_ values ranged from 183.26 μM to 68.07 μM relative to the lung cancer cell line and in the range of 68.41–19.70 μM relative to the colon cancer cell line. The strongest effect against A549 was demonstrated by compound 4 (Fig. 3), and against the HT29 cell by molecule 6 (Fig. 4). Therefore, they were chosen for research on human cells.

Control compounds showed significantly lower cytotoxic activity than the tested derivatives—IC_50_ etoposide 451.47 μM and 654.03 μM, 5-fluorouracil IC_50_ 1626.85 μM and > 1800 μM. For the A549 cell line, IC_50_ values were higher than for HT29 cells, indicating that colon adenocarcinoma cells are more sensitive to tetrahydroacridine derivatives than lung adenocarcinoma cells. The compounds were divided into two groups, depending on the position of the fluoride. Against lung and colorectal carcinoma, the structure–activity relationship showed that a longer carbon linkage and fluoride substitution in meta, and preferably in a pair, positions significantly increased cytotoxic activity.

**Fig. 5:**
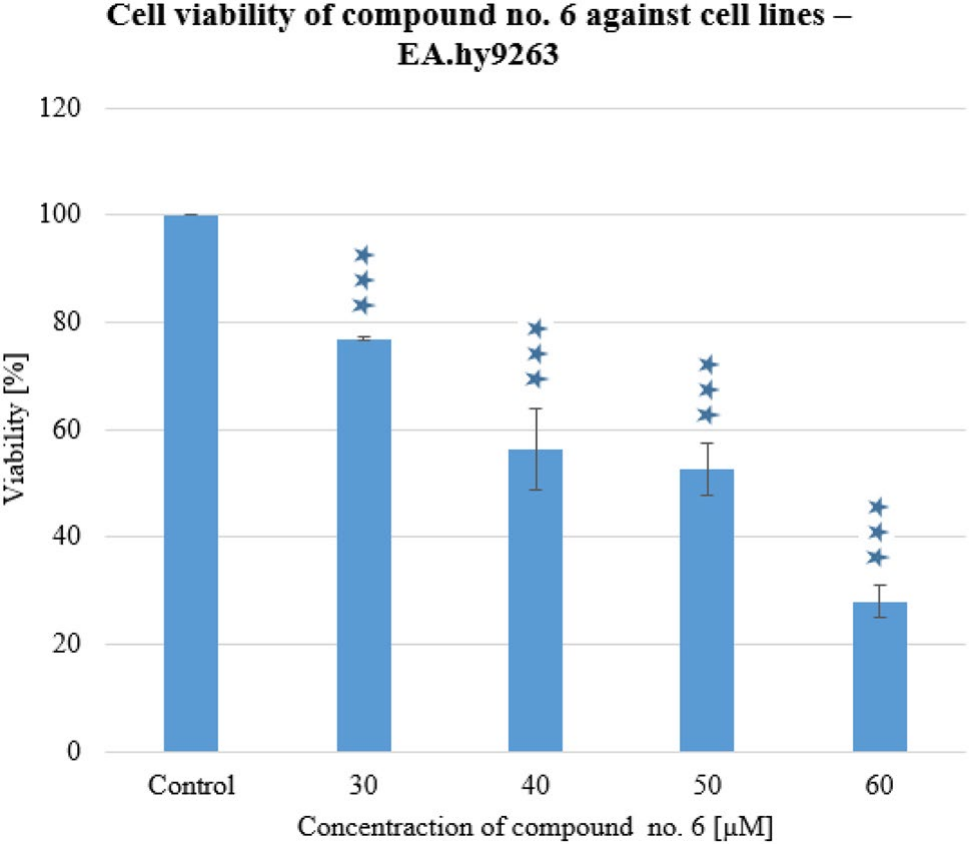
Cell viability of compound no. 6 against cell lines—EA.hy9263. Experiments were done in triplicates. Statistical significance was assessed using one-way ANOVA with a post-hoc analysis was performed. ***p < 0.001, **p < 0.01, and *p < 0.05 were considered as significantly different in comparison to non-treated control

The cytotoxicity effects on EA.hy926 cells were comparable for both compositions and were approximately 50 μM. There was a significant difference between cancer and non-cancer cell line IC_50_ results. These results suggest that the compounds were more selective for colon cancer cells than normal cells. Compound **6** (Fig. 5) was slightly more toxic than compound **4**. Etoposide showed a higher cytotoxic activity on EA.hy926 cells (IC_50_ 155.19 μM) than A549 cells (IC_50_ 451.47 μM) that showed toxic effects on endothelial cells. While 5-fluorouracil exhibited similar cytotoxicity to HT29 and EA.hy926 cell lines.

### In vitro inflammation study on HYAL

Hyaluronidase is an enzyme that undergoes depolymerization of hyaluronan (part of the extracellular matrix) and thus the integrity of the tissue is attenuated during the inflammatory process. This is the reason for the interest of many scientists studying new drugs with additional anti-inflammatory properties [27–30].

In the in vitro inflammation study on HYAL, inhibitory activity of new compounds was determined by spectrophotometric method. All compounds tested showed significant inhibitory activity. The IC_50_ values ranged from 52.27 to 272.31 μM, which with positive control (heparin) are shown in Table 2.

**Table 2:** In vitro hyaluronidase inhibitory activity of new compounds and a positive control, heparin

Moiety	2-Fluorobenzoic acid			3-Fluorobenzoic acid			Positive control—heparin
No	**1**	**2**	**3**	**4**	**5**	**6**	**C3**
Number of methylene groups	2	3	4	2	3	4	–
IC_50_ (µM)	244.26***	217.94***	192.84***	52.27***	244.98***	272.31***	56.41
SD	± 4.40	± 2.24	± 1.92	± 0.58	± 1.55	± 1.99	0.78

All values are presented as the means ± standard deviation (SD)

Compound 4 (bold font) had the best inhibitory activity

Statistical significance was assessed using one-way ANOVA

*IC*_*50*_ 50% inhibition of enzyme activity, *µM* micromole/liter

***p < 0.001 was considered as significantly different from control heparin

The inhibitory activity among compounds was influenced by the fluorine position and the number of methylene groups. For 2-fluorobenzoic acid derivatives, the higher number of methylene groups accelerated the inhibition, while for 3-fluorobenzoic acid the reverse situation was observed. Compound **4** had the best inhibitory activity (IC_50_ 52.27 µM), comparable to the positive control (IC_50_ 56.41 µM). It can be concluded that tetrahydroacridine derivatives have some anti-inflammatory activity.

### In silico assessment of toxicity

The estimation of toxicity of recently synthesized pharmacological active compounds is one of the most important part of the investigation of new drugs. At this early stage of research, low-cost and fast methods based on in silico mathematical calculations are very useful. In this study, a well-known software was used to predict the acute toxicity, mutagenicity and carcinogenicity of the new synthesized compounds (Table 3).

**Table 3:** In silico predicted acute toxicity, mutagenicity and carcinogenicity of studied new tetrahydroacridine derivatives

Moiety	2-Fluorobenzoic acid			3-Fluorobenzoic acid		
No	**1**	**2**	**3**	**4**	**5**	**6**
LD_50_ (Mouse IP)	400	170	160	400	170	160
LD_50_ (Mouse OR)	1300	630	540	1300	630	540
LD_50_ (Mouse IV)	65	52	48	65	52	48
LD_50_ (Mouse SC)	400	360	240	400	360	240
LD_50_ (Rat IP)	180	200	160	180	200	160
LD_50_ (Rat OR)	1200	790	680	1200	790	680
Mutagenicity	0.74	0.66	0.67	0.74	0.66	0.67
Carcinogenicity	0.07	0.33	0.33	0.07	0.33	0.33

*IP* intraperitoneal, *OR* oral, *IV* intravenous, *SC* subcutaneous, LD_50_ [mg/kg]

Percepta software was used to estimate the acute toxicity to rodents on six models: mice intraperitoneal, mice oral, mice intravenous, mice subcutaneous, rat intraperitoneal and rat oral. The obtained results expressed in LD_50_ values clearly show that the tested tetrahydroacridine derivatives are slightly toxic compounds and LD_50_ [mg/kg] values ranged from 680 to 1200 (rat oral model). It can also be observed that the acute toxicity of the studied compounds increases with the rise of the atoms of carbon in aliphatic chain.

The same software was also used for the prediction of mutagenicity and estimation of the probability of positive Ames test. The obtained results indicate that the analyzed compounds possess low mutagenic potential (probability ranged 0.66–0.74) and the differences between the derivatives are insignificant.

Vega software was used to predict the carcinogenicity of the studied tetrahydroacridines and a very low probability of carcinogenic activity (0.07–0.33) was obtained. It should be also noticed that the position of fluorine in fluorobenzoic moiety has no influence on the carcinogenic potential as well as mutagenic and acute toxicity of the studied compounds.

### Acute oral toxicity: acute toxic class method

Two best-performing compounds, i.e. no. **4** and **6**, were tested.

For substance no. **6**, 300 mg/kg was given to 2 groups. After first dose of 300 mg/kg, no mouse died, and after second dose of 300 mg/kg, 1 mouse died. Then, dose 2000 mg/kg was given. In this step, all 3 mice died. Therefore, dose 2000 mg/kg was not repeated for the second time and compound can be classified in category 4 of GHS scale, with LD_50_ cut-off 500 mg/kg. This result was comparable with in silico assessment of toxicity—compound **6** had LD_50_ value of 540 mg/kg.

After first dose of substance No. **4** of 300 mg/kg, 2 mice died. Therefore, this dose was not repeated and lower dose, 50 mg/kg, was given to the mice. In this step, 1 mouse died. Dose 50 mg/kg was repeated and none mouse died. Therefore, compound can be classified in category 3 of GHS scale, with LD_50_ cut-off 300 mg/kg. In the in vivo test, compound **4** turned out to be much more toxic than in silico assessment (LD_50_ value of 1300 mg/kg).

**Fig. 6:**
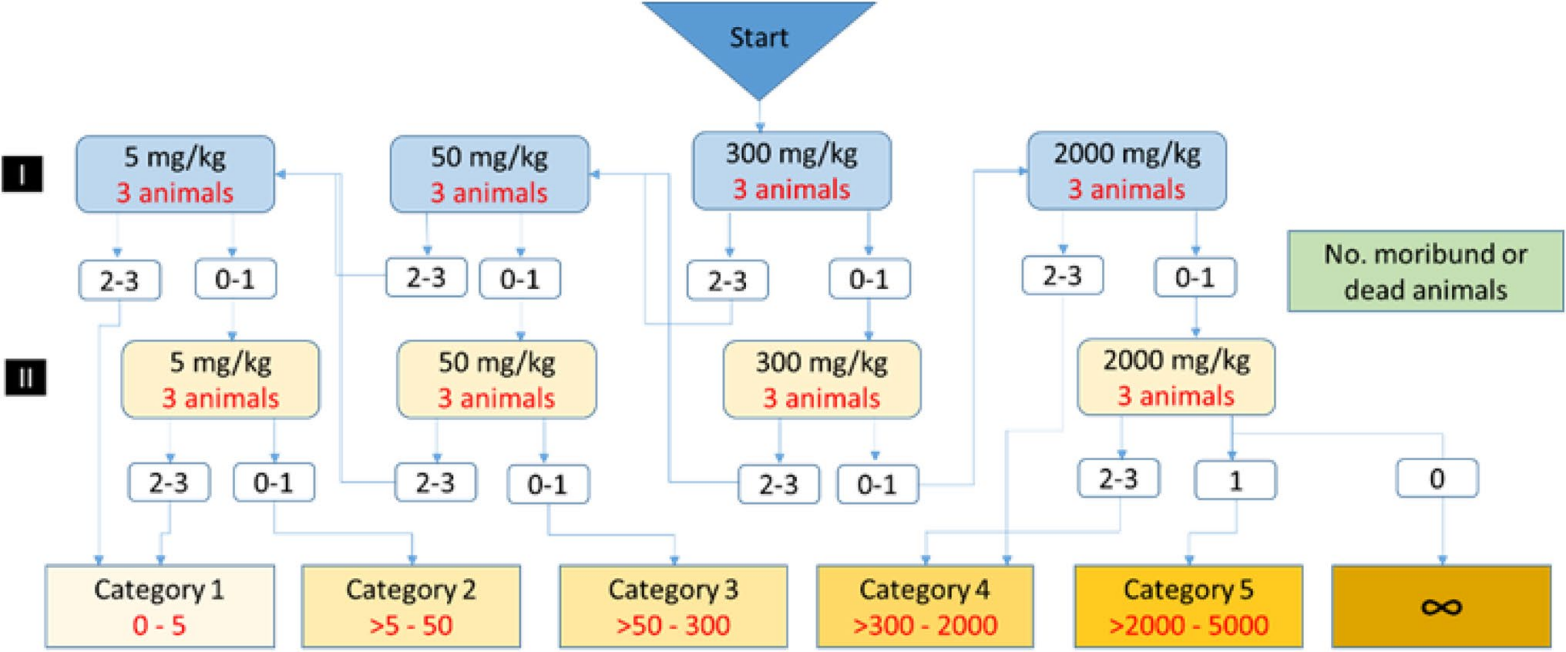
Classification scheme of acute oral toxicity

As a result of the tests, it can be concluded that substance no. **6** can be classified as category 4, while substance no. **4** can be classified as category 3 (Fig. 6).

## Conclusion

The aim of the study was to determine the cytotoxic activity of six new tetrahydroacridine derivatives with a fluorobenzoic moiety on human cell lines A549 (lung adenocarcinoma), HT-29 (colon adenocarcinoma) and EA.hy926 (hybrid umbilical vein). The results confirmed cytotoxicity activities of novel compounds. Moreover, all compounds presented higher cytotoxicity activity than the reference drugs—etoposide and 5-fluorouracil. HT29 cells were more sensitive to the novel compounds than AA549 cells and the normal cell line. It can be concluded that novel compounds might be used in the treatment of colorectal cancer, with less harm to the vein cells and stronger toxicity toward cancer cells. Among all derivatives, compounds **4** and **6** showed the highest activity. Both belong to the derivatives with position of fluorine in *meta* position, which could indicate higher activity of compounds with substitution in *meta* than in *ortho* position. The anti-inflammatory activity was determined in the hyaluronidase inhibition assay. Compound **4** showed the highest inhibitory activity, what is more, slightly higher than a positive control. Compounds confirmed their inhibitory activity and in result, anti-inflammatory properties. Comparing results from the studies, compound **4** turned out to be the best compound to further studies on lung cancer, whereas compound **6** might be considered in the research against colorectal cancer. Therefore, these compounds were tested on animals. The results of tests carried out on the mouse model in accordance with OECD 423 allowed to qualify the substance no. **6** to category 4 of the GHS scale and substance no. **4** to category 3 of the GHS scale. The mathematical calculations showed that the tested tetrahydroacridine derivatives are slightly toxic compounds and the LD_50_ values [mg/kg] ranged from 680 to 1200 (oral rat model), the analyzed compounds have low mutagenic potential (the probability ranged from 0.66 to 0.74), and differences between derivatives are insignificant and very low probability of carcinogenicity (0.07–0.33).
